# Can Acute Postoperative Pain Management After Tumour Resection Surgery Modulate Risk of Later Recurrence or Metastasis?

**DOI:** 10.3389/fonc.2021.802592

**Published:** 2021-12-16

**Authors:** Aneurin Moorthy, Aisling Ní Eochagáin, Donal J. Buggy

**Affiliations:** ^1^ Anaesthesiology & Perioperative Medicine Research Fellow, Division of Anaesthesiology and Peri-operative Medicine, Mater Misericordiae University Hospital, Dublin, Ireland; ^2^ Anaesthesiology Research Fellow, St. James’s University Hospital, Dublin, Ireland; ^3^ Consultant and Professor, Division of Anaesthesiology and Peri-operative Medicine, Mater Misericordiae University Hospital, School of Medicine, University College, Dublin, Ireland; ^4^ Outcomes Research, Cleveland Clinic, Cleveland, OH, United States

**Keywords:** acute pain, cancer, cancer recurrence, metastasis, anaesthesia

## Abstract

**Background:**

Cancer is a leading cause of mortality worldwide, but death is rarely from the primary tumour: Rather it is multi-organ dysfunction from metastatic disease that is responsible for up to 90% of cancer-related deaths. Surgical resection of the primary tumour is indicated in 70% of cases. The perioperative stress response, tissue hypoxia at the site of surgery, and acute pain contribute to immunosuppression and neo-angiogenesis, potentially promoting tumour survival, proliferation, and metastasis. Poorly controlled acute postoperative pain decreases Natural Killer (NK) immune cell activity, which could potentially facilitate circulating tumour cells from evading immune detection. This consequently promotes tumour growth and distal metastasis.

**Methods:**

We conducted a comprehensive literature search for links between acute pain and cancer outcomes using multiple online databases. Relevant articles from January 1st, 2010 to September 1st, 2021 were analysed and appraised on whether postoperative pain control can modulate the risk of recurrence, metastasis, and overall cancer survival.

**Results:**

Although experimental and retrospective clinical data suggest a plausible role for regional anaesthesia in cancer outcome modulation, this has not been supported by the single, largest prospective trial to date concerning breast cancer. While there are mixed results on anaesthesiology drug-related interventions, the most plausible data relates to total intravenous anaesthesia with propofol, and to systemic administration of lidocaine.

**Conclusion:**

The hypothesis that anaesthetic and analgesic technique during cancer surgery could influence risk of subsequent recurrence or metastasis has been prevalent for >15 years. The first, large-scale definitive trial among women with breast cancer found robust equivalent findings between volatile anaesthesia with opioid analgesia and regional anaesthesia. Therefore, while regional anaesthesia during tumour resection does not seem to have any effect on cancer outcomes, it remains plausible that other anaesthetic techniques (e.g. total intravenous anaesthesia and systemic lidocaine infusion) might influence oncologic outcome in other major tumour resection surgery (e.g. colorectal and lung). Therefore, another large trial is needed to definitively answer these specific research questions. Until such evidence is available, perioperative analgesia for cancer surgery of curative intent should be based on patient co-morbidity and non-cancer endpoints, such as optimising analgesia and minimising postoperative complications.

## Introduction

In 2020, it was estimated that 18 million new cancer cases were diagnosed, (excluding nonmelanoma skin cancer). This was associated with approximately 10 million cancer related deaths (1). The incidence of female breast cancer has exceeded lung cancer and is now the most prevalent cancer among women. Furthermore, it is estimated that by 2040, the global overall cancer burden will rise by 47%, which approximates to 28 million cases (1). The value of surgery in the treatment of solid tumours is evident, because they are amenable to surgical resection. Surgery offers the best chance of a cure and improves prognosis. This is particularly true for early-stage disease (2). Metastasis is defined as a complex multistep process in which tumour cells disseminate from the primary neoplasm to secondary sites (3). The primary tumour is rarely the cause of death for cancer patients. In reality, the metastatic process and resultant organ dysfunction is accountable for 70-90% of cancer related deaths (4, 5).

Minimal residual cancer is defined as an undetectable group of malignant cells that persist after surgical resection (6). This occurs as a result of inadequate surgical clearance, incomplete surgical margins or seeding of cancerous cells into the surgical field, blood or lymphatic system during the intraoperative period. Alternatively, these cells may already exist prior to surgery as subclinical micro-metastatic disease. Survival of these cancerous cells depends on an array of factors, such as surgical stress response, tissue hypoxia, inflammation, and pain. All of these elements suppress the immune system during cancer surgery. Therefore, host immunosuppression will assist these tumour cells to escape cellular destruction and thus aid metastasis (7). Additionally, other factors such as perioperative blood transfusion, hypothermia, and more aggressive cancer types may negatively influence the risk of cancer recurrence (8, 9). Analgesic agents are used along with both general and regional anaesthesia techniques during surgery, to obtund the surgical stress response and manage perioperative pain. Moreover, a large number of preclinical and experimental data over the past 30 years have suggested that various anaesthetic and analgesic agents may exhibit potentially beneficial cancer-resisting effects, while others may demonstrate potentially harmful cancer-promoting effects (10). The perioperative period during cancer surgery is a critical time of immunological susceptibility. Therefore, anaesthetic and analgesic techniques may have a role in modulating this risk, consequently potentially affecting postoperative oncologic outcomes (11). In the past few decades, only one high quality randomised controlled trial has been conducted to test this hypothesis. In this review article, we will explore how sub-optimal management of acute perioperative pain may be associated with cancer recurrence, and whether or not common analgesic agents and strategies used during the perioperative period may influence the risk of cancer recurrence or metastasis.

## Methods

A literature search for links between acute pain and cancer outcomes was conducted using the following databases: Medline/Pubmed, EMBASE, Google Scholar, Cochrane Central Register of Controlled Trials (CENTRAL), Web of Science, and CINAHL. Key search terms such as ‘acute pain’; ‘cancer’; ‘cancer recurrence’; ‘regional anaesthesia and cancer’; ‘postoperative analgesia and cancer recurrence’; ‘analgesia and metastasis’; ‘opioids and cancer recurrence’, and ‘perioperative pain control’, were used to analyse the relevant literature. Studies from 1 January 2010 until 1 September 2021 were included. This comprised of randomised controlled trials, retrospective studies, meta-analyses, systematic reviews, relevant review articles and any referenced articles deemed important regardless of the publication date. Articles were assessed for importance and significance by all named authors. For the purpose of this review article, we included what were, in our opinion, the most notable, relevant and recent data.

## Pain and Cancer Recurrence

At an anatomical level, cancer is made up of tumour cells surrounded by the tumour microenvironment. This microenvironment consists of an extracellular matrix, blood vessels and various host cells (fibroblasts, mesenchymal and various immune cells) (12). Additionally, a subset of tumour cells called ‘cancer stem cells’, that play an important role in facilitating tumour metastasis, are found within this environment (13). Cancer surgery can easily disrupt this environment and inevitably may promote spread of residual cancer cells. Postoperative cancer recurrence may occur *via* the following mechanisms (8):

1. Local recurrence at the surgical resection site.
2. Lymph node metastasis.
3. Secondary organ metastasis as a result of circulating tumour cells (CTCs) seeding before or during the perioperative period.

The likelihood for CTCs to survive and lodge in distant tissues during the perioperative period is not fully understood, but can be influenced by numerous immunomodulating factors. These include pain, surgical stress response, and degree of inflammation caused by the surgery itself (10). Interleukin (IL)-6, IL-1-beta, tumour necrosis factor (TNF)-alpha, and vascular endothelia growth factor (VEGF) are important inflammatory mediators that are released during surgery and the postoperative period. These all have significant implications in survival of residual cancer cells (14). Moreover, distal inflammatory sites may provide the ideal site for CTCs to collect during the perioperative period, a process called inflammatory oncotaxis (15). In addition, the inflammatory response depresses the host immune function by impairing natural killer (NK) cells cytotoxicity (16). NK cells are particularly important in preventing tumorigenesis and metastasis (17). The surgical stress response results in activation of the sympathetic and neuroendocrine system to stimulate the release of catecholamines and cortisol. Again, this impairs the immune system by inhibiting the antitumour activity of NK cells and CD8+T cells. These humoral factors promote the proliferation of T regulatory and Type 2 helper T cells (Th2), which supports cancer cell growth (18). Early laboratory data has demonstrated that surgical trauma increases host susceptibility to experimental metastasis formation (19, 20). The impaired immune system, in particular cell mediated immune function, can result in circulating tumour cells evading host detection. Therefore, it is plausible to speculate that the stronger and more uncontrolled the surgical stress response is, the greater the risk of distal metastasis occurring during the perioperative period.

**Figure 1:**
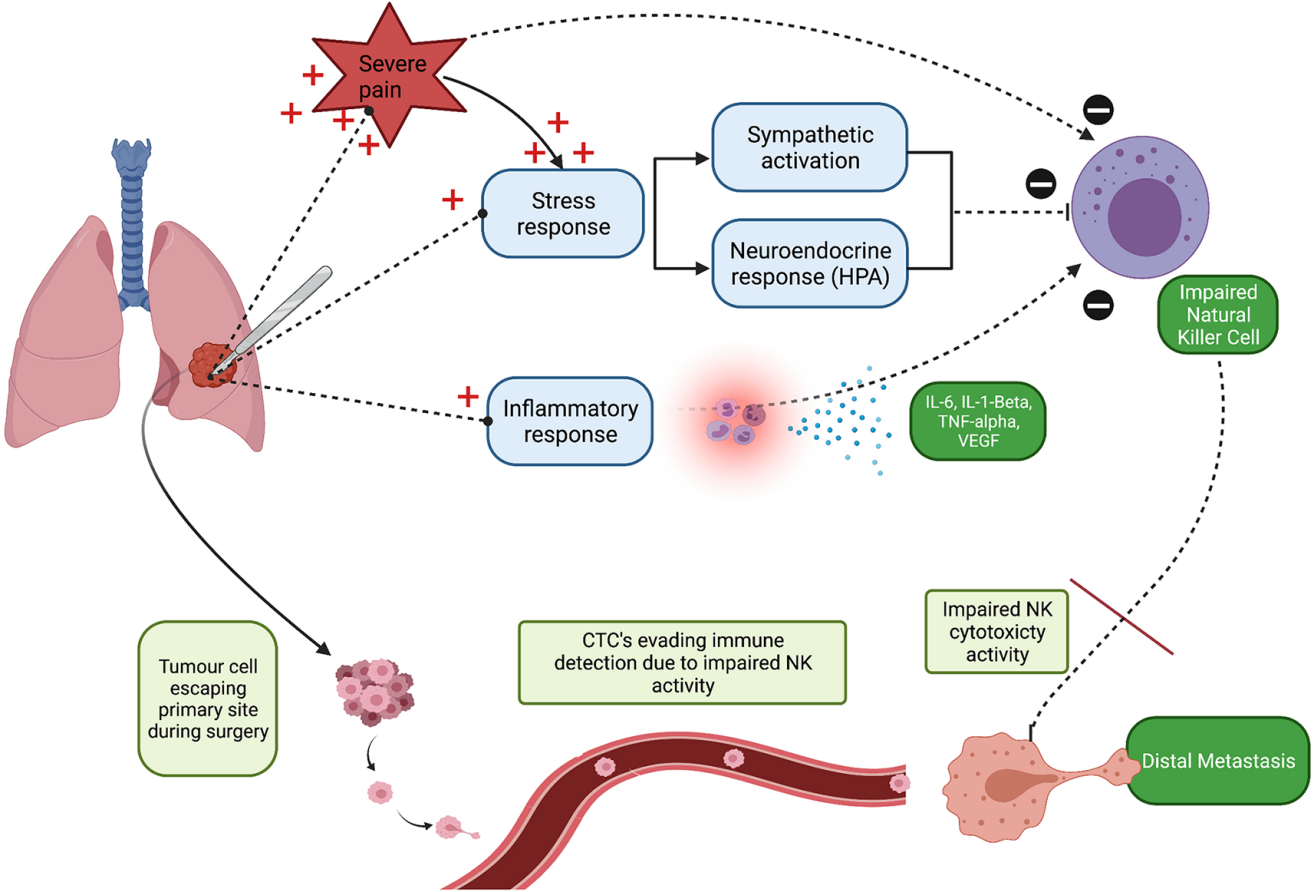
Schematic illustration highlighting that poorly controlled acute perioperative pain may promote tumour cell seeding and consequently increase the risk of distal metastasis. NK, Natural Killer cell; CTCs, Circulating Tumour Cell’s. Created with BioRender.com.

Pain is defined as ‘an unpleasant sensory and emotional experience associated with actual or potential tissue damage’ (21). It is a multidimensional experience and personalised to each patient (22). Acute pain refers to pain that does not persist for longer than three months (22). Acute perioperative pain is a consequence of surgical trauma, inflammation, and sympathetic system over-reactivity, the latter being an important factor that contributes to the transition from acute to chronic persistent post-surgical pain (23). Animal experimental data consistently suggests that poorly controlled pain following surgical trauma promotes postoperative immunosuppression, and, in turn may enhance malignant processes (24–26). The most notable immunosuppressive effect demonstrated in these studies was decreased NK cell count and activity (24–26). Moreover, uncontrolled acute perioperative pain may exacerbate the surgical stress response, due to enhanced activity to both the sympathetic nervous system and neuroendocrine responses. Therefore, this may additionally increase the risk of postoperative cancer recurrence/metastasis by further decreasing NK cell activity. This sequence of events is summarised in **Figure 1**.

Theoretically, satisfactory acute perioperative pain control and associated obtundation of the surgical stress response may potentially reduce cancer recurrence risk. A recent systematic review and meta-analysis of experimental animal data compared the risk of cancer metastasis between two groups, analgesic versus control treatment. The authors suggested that analgesics, in particular NSAIDs, significantly reduce the risk of metastasis in various animal models (n=7,000) (27). However, translatability of these experimental findings (27) to the clinical situation remains unclear. It would undoubtedly be unethical to test this hypothesis in a prospective, randomised control trial in patients undergoing cancer surgery. It would involve purposely withholding effective analgesia strategies in one group and not in the other. Instead this is limited to retrospective data, a (28) retrospective review of 2,401 patients who underwent colorectal cancer resection included 13,931 pain score observations. Results showed that approximately 10% of these surgical patients had persistent moderate to severe pain up to five days postoperatively. This group had the highest risk of cancer recurrence and mortality when compared to patients from the same cohort who only experienced mild postoperative pain (28).

**Figure 2:**
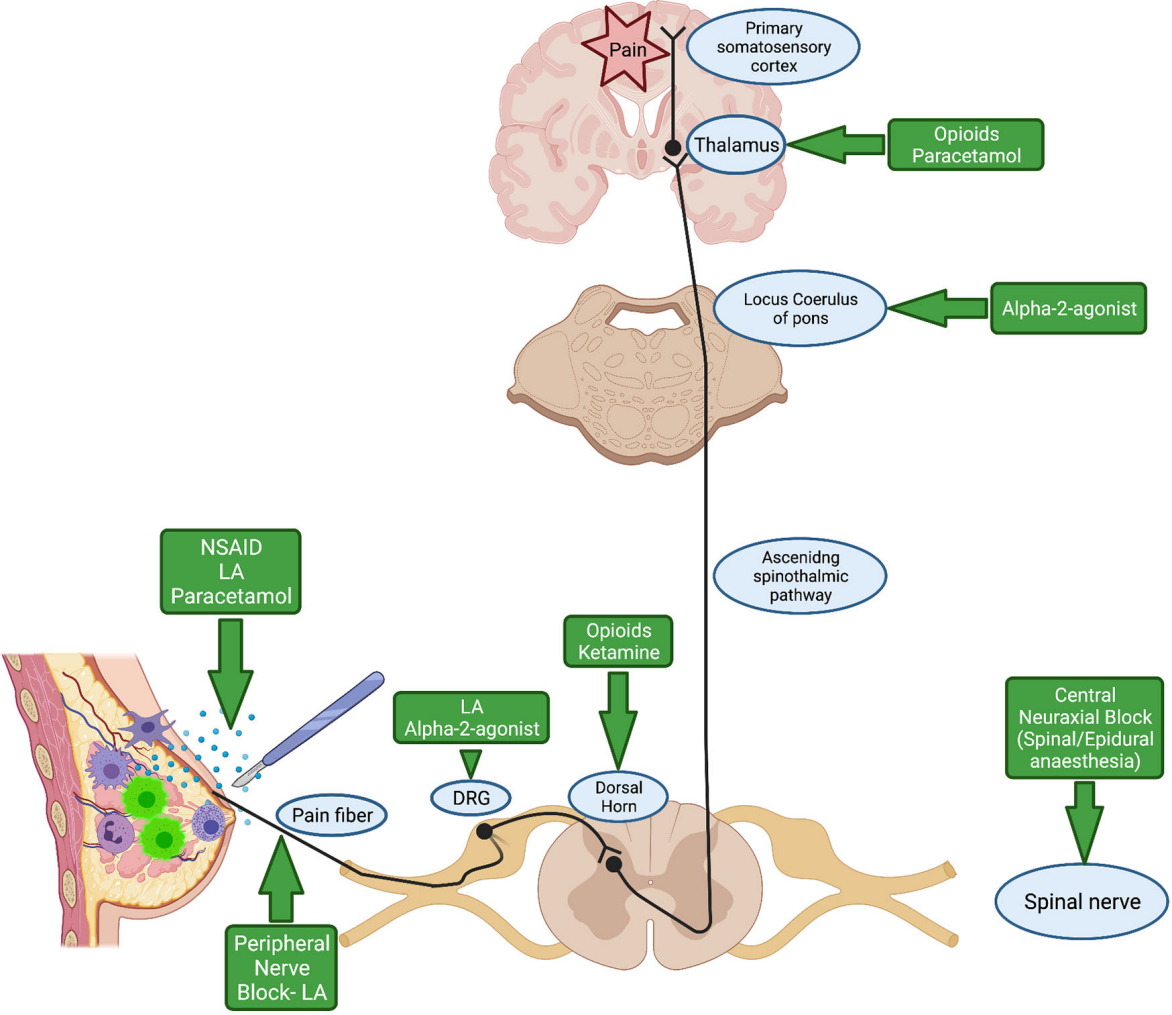
Pain pathway and site of action of commonly used analgesic agents during the perioperative period. NSAID, Non-steroidal anti-inflammatory drug; LA, Local Anaesthetic; DRG, Dorsal Root Ganglion. Created with BioRender.com.

**Figure 2** illustrates the pain pathway and site of action of common analgesia adjuvant agents used during the perioperative period. We will review each of these analgesic agents used during cancer surgery and summarise the current evidence relating to their effect on potential cancer recurrence.

## Regional Anaesthesia: Acute Pain Management and Cancer Recurrence

Regional anaesthesia is defined as applying local anaesthetic agents to an individual nerve, plexus of nerves, or to an anatomical plane through which nerves pass, in order to render a distal site anaesthetised (29). Use of regional anaesthesia techniques is increasing worldwide. In the operating theatre, regional anaesthesia can be used solely to achieve surgical anaesthesia, and may also be used to complement general anaesthesia to effectively manage acute pain and postoperative recovery after various types of surgery (30, 31). There are many different regional anaesthesia techniques including: spinal anaesthesia, epidural anaesthesia, fascial plane blocks and peripheral nerve blocks (32)

Local anaesthetic agents are the principal drugs used in regional anaesthesia procedures. These drugs are water-soluble salts, or lipid soluble alkaloids, and are made up of three structures: a hydrophilic amine group, a hydrocarbon link and a lipophilic aromatic group (33). Local anaesthetic agents are categorised into esters or amides, depending on the structure of this hydrocarbon intermediary link chain (33). *In vitro* experiments involving ropivacaine, an amide local anaesthetic agent, have demonstrated antimetastatic effects by inhibiting migration of cancer cells (34) and interfering with cell differentiation and tumorigenesis (35).

Lidocaine is an amide local anaesthetic agent and is commonly used during cancer surgery (36). It contains potent analgesic, anti-hyperalgesia and anti-inflammatory properties (36). Alternatively, an additional benefit to lidocaine’s analgesic effects, are its potential antitumour properties. Therefore, it has been suggested that the use of lidocaine during and after surgery could improve oncological outcomes, by reducing the ability of cancer cells to recur and metastasise (37). The anticancer effects of lidocaine have been extensively demonstrated in multiple *in vitro* studies. At various concentrations (0.1 mM-10 mM), it manifests antitumour effects by inhibiting proliferation (38, 39), migration (39, 40) and invasion of cancer cells (39), and by inducing cell cycle arrest (41). Lidocaine’s inhibitory action on voltage-gated-sodium-channels plays a significant role in the process of cancer metastasis (42). In addition, at clinically relevant doses, lidocaine has been shown to demonstrate anti-DNA tumour replication activity in oestrogen receptor negative and positive breast cancer cell lines (43). Furthermore, *in vivo* studies have indicated that lidocaine inhibits metastasis in murine cancer models by multiple mechanisms (44–48). It also appears that lidocaine has a greater affect at attenuating the development of pulmonary metastasis as compared to other organ sites. **Table 1** summaries the findings of the most recent animal experiments on lidocaine and its effect on cancer metastasis.

**Table 1:** Selected summary of recent *in vivo* studies investigating the antitumour effects of lidocaine.

Author	Year	Lidocaine dosage	Finding	Proposed mechanism of action
Freeman et al. (44)	2018	1.5mg/kg bolus followed by a 30-40 minute infusion at 2mg/kg/hr	lidocaine combined with Cisplatin significantly decreased metastatic lung colony count in a murine model of breast cancer surgery.	Lidocaine enhanced the metastasis-inhibiting action of cisplatin.
Goa et al. (45)	2018	Co-loading of lidocaine and cisplatin by ligand-modified nanogels.	Targeted delivery of co-loaded lidocaine and cisplatin inhibited the primary tumour growth but also alleviated lung metastasis.	Co-loaded lidocaine and cisplatin by ligand-modified nanogels exhibited higher selective cellular uptake and enhanced the apoptosis activity of cisplatin.
Johnson et al. (46)	2018	Combination of 1.5mg/kg lidocaine bolus followed by 25 minute infusion at 2mg/kg/hr and inhalational sevoflurane during the perioperative period.	Lidocaine reduced lung metastatic colony count and proportion of pulmonary metastasis versus sevoflurane inhalational anaesthesia alone in a murine model of breast cancer.	Reduced anti-inflammatory and anti-angiogenic effects when lidocaine was introduced.
Wall et al. (47)	2019	1.5mg/kg bolus followed by a 25 minute infusion at 2mg/kg/hr	Lidocaine reduced pulmonary metastasis in a murine model of breast cancer surgery model but was ineffective against liver metastatic colonies	Inhibitory effect on Matrix Metallopeptidase 2.
Liu et al. (48)	2021	Intraperitoneal injection of (0.5%, 50 μl) lidocaine into murine model once a day for three days	Lidocaine retarded the metastasis and induced apoptosis in ovarian cancer tissues of a murine ovarian cancer model.	Lidocaine blocked the NaV1.5 channel and subsequently malignancy through inactivation of FAK/Paxillin signalling pathway

Unfortunately, the translation of these laboratory findings to the clinical setting is limited (44–48). Zhang Hao et al. (49) conducted a retrospective study of 2,239 patients who underwent pancreatectomy for pancreatic cancer. They reported that the use of intraoperative intravenous lidocaine infusion was associated with improved overall survival, but not disease-free survival, compared to the non-lidocaine group.

Neutrophil extracellular trapping (NETosis) is a process where neutrophils degranulate when exposed to tumour antigens, and is a potential biomarker for metastatic risk (50). A randomised controlled trial investigated the addition of intravenous (IV) perioperative lidocaine during breast cancer surgery, and concluded that IV lidocaine decreased postoperative expression of NETosis, therefore potentially reducing the rate of cancer recurrence (51).

Large prospective, well-designed, randomised controlled clinical trials are urgently needed to assess the protective effect of lidocaine on recurrence after cancer surgery to achieve a “proof of concept”. At present, the VAPOR-C Trial (Volatile Anaesthesia and Perioperative Outcomes Related to Cancer, NCT04316013) aims to accomplish this. This large, multicentre trial is a pragmatic randomised controlled trial, with a 2x2 factorial design, comparing volatile anaesthesia with sevoflurane versus total intravenous anaesthesia with propofol. Within these two arms, patients will be further randomised to receive perioperative lidocaine according to standard use, or saline control. The study aims to enrol a total of 5,763 participants globally, with its primary outcome being disease free survival. A feasibility and pilot study were recently completed (52). The authors demonstrated a successful adherence to randomisation in 99.3% of their study cohort. Recruitment for VAPOR-C has begun, and its’ estimated completion date is 2027. In addition, the ‘ALLEGRO RCT (ISRCTN 52352431), another ongoing multicentre RCT, will examine the effect of Intravenous lidocaine bolus followed by an infusion during colorectal cancer surgery. Cancer outcomes up to 10 years post patient surgery will be assessed.

Regional anaesthesia offers numerous benefits during the peri-operative period. These include superior analgesia, reduced length of hospital stay, improved quality of early recovery and fewer postoperative cardiorespiratory complications (30, 53, 54). Moreover, regional anaesthesia-analgesic regimes attenuate the surgical stress response and diminishes the amount of opioids required during the perioperative period (55, 56). As discussed below, the findings of some laboratory and preclinical studies suggest that opioids may be associated with immunosuppressive properties and thus promote tumorigenesis. Impaired host resistance may increase the risk of cancer metastasis during the perioperative period. Experimental data from murine models have suggested that perioperative pain control may play a crucial role in preventing impairment in host resistance after surgery (24). It has been postulated that incorporating regional anaesthesia regimes into cancer surgery to provide excellent perioperative analgesia and to blunt the surgical stress response, may have a role in modulating the risk of cancer recurrence or metastasis. An original retrospective review conducted by Exadaktylos and colleagues, suggested an association between paravertebral anaesthesia and analgesia for breast cancer surgery and a reduced risk of metastasis (57). However, the first multicentre randomised controlled trial on the effect of anaesthetic and analgesic techniques on long term oncologic outcome, published in The Lancet by our group (58) demonstrated robust equivalent findings, regardless of anaesthetic technique. Over 11 years (2007–2018), the authors randomised 2,132 patients to receive either regional anaesthesia-analgesia (paravertebral combined with propofol IV general anaesthesia), or general anaesthesia (sevoflurane) and opioid analgesia. The rate of cancer recurrence between the two groups was similar, at approximately 10%. We concluded that paravertebral regional anaesthesia-analgesia did not reduce cancer recurrence after intended curative surgery.

Neuraxial anaesthesia includes both epidural and spinal anaesthesia procedures. Both techniques are widely used for acute pain management after thoracic and abdominal cancer surgeries. Epidural analgesia is achieved by placing an epidural catheter into the epidural space, which is used to administer a continuous infusion of local anaesthetic agents with or without opioids into this space. This catheter is usually left *in situ* for up to four days to achieve satisfactory analgesia in the early postoperative period when acute pain is most intense. The catheter is not left in the epidural space for longer than four days as the risk of infection significantly increases beyond this time frame (59). In contrast, spinal anaesthesia involves a single dose of local anaesthetic, usually 15-20mg of bupivacaine/Levobupivacaine with/without opioids administered into the intrathecal space. This provides surgical anaesthesia and analgesia for up to 6 hours (60).

Numerous retrospective studies have been performed to determine if there is an association between neuraxial anaesthesia and cancer recurrence. The results from these reviews are conflicting. To date there have been five meta-analyses conducted to answer this question. These meta-analyses dated between 2014 and 2020 (61–65). **Table 2** summarises the findings from these meta-analyses. These retrospective analyses suggests that perioperative neuraxial anaesthesia techniques may be associated with an improved overall survival in patients undergoing cancer surgeries, especially for colorectal and prostate cancer. However, the majority of these studies failed to demonstrate a decrease in cancer recurrence rates.

**Table 2:** Summary of recent meta-analysis of neuraxial anaesthesia and cancer recurrence.

Author	Year	Regional anaesthesia	Total Number of studies analysed	Findings
Lee et al. (65)	2020	Epidural and paravertebral anaesthesia	6 (3,139 patients in the regional anaesthesia group)	Adjunctive use of epidural or paravertebral anaesthesia with general anaesthesia did not reduce the rate of cancer recurrence following cancer surgery.
Weng et al. (61)	2016	Epidural and Spinal anaesthesia	20 (15,160 patients in regional anaesthesia group)	Neuraxial anaesthesia appears to improve overall survival, specifically in colorectal cancer surgery and may be associated with reduced risk of cancer recurrence.
Sun et al. (62)	2015	Epidural and Spinal anaesthesia	20 (16,618 patients in regional anaesthesia group)	Perioperative neuraxial anaesthesia may improve overall survival after cancer surgery but it had no positive influence in the reduction of cancer recurrence.
Lee et al. (64)	2015	Epidural and Spinal anaesthesia	10 (7,504 patients in regional anaesthesia group)	Neuraxial anaesthesia during prostate cancer surgery appears to improve overall survival but was not associated with longer recurrence-free-survival.
Pej et al. (63)	2014	Epidural anaesthesia	10 (3,254 patients in regional anaesthesia group)	Perioperative epidural anaesthesia did not influence postoperative cancer recurrence and metastasis rate. However, epidural anaesthesia may be associated with improvement in prognosis of prostate cancer surgery with a follow-up of less than or equal to two years.

A recent small RCT aimed to investigate the effect of epidurals on cancer recurrence. The authors randomised 400 patients undergoing lung cancer surgery, to receive a combined epidural-general anaesthetic or a general anaesthetic with opioid analgesia. This trial was adequately powered to detect a relative reduction in cancer recurrence. The authors concluded that the insertion of an epidural as an adjuvant to general anaesthesia and for acute postoperative pain management, did not improve cancer recurrence rate and overall survival, for patients undergoing lung cancer surgery compared to general anaesthesia alone (66).

## Opioids

Opioids are primarily used for cancer patients to provide analgesia in both the acute and chronic settings. While they have known beneficial analgesic properties, they also have non-analgesic effects, including direct and indirect effects on cancer cells. Laboratory studies have investigated numerous mechanisms by which opioids may influence cancer cells, however, results of these studies are inconsistent. Clinical studies investigating perioperative opioids have not shown consistent links between their use and increased risk of tumour growth and metastasis (58).

Preclinical studies have investigated the effects of opioids on immunosuppression and inflammation. Opioids have been shown to have direct and indirect effects on cancer cells and on anti-tumour immunity, (NK cells, macrophages and T-cells). Direct effects on immune cells are materialised *via* opioid and non-opioid toll-like receptors. Cancer cells show an overexpression of μ opioid receptors (MOR), therefore opioids may directly influence their growth (67). MOR overexpression has been linked with the development of metastases in patients with lung, prostate and oesophageal cancer (67–69). Subclasses of opioids have been shown to have varying effects on cancer cells; specifically morphine has been shown to influence the proliferation and survival of cancer cells *via* direct effects on tumour cell DNA cleavage, Akt, PlK, MAPK, Src, GRB2-associated binding protein 1 (Gab-1) and STAT3 signalling pathways (70–72). A study of patients with breast cancer found that those with an MOR gene polymorphism had reduced cancer-related mortality over a ten-year period (73). Methylnaltrexone, which is a MOR antagonist, has shown consistent findings in the role of MOR in cancer progression, in that it may have beneficial effects in stopping cancer progression and metastasis. In the laboratory setting, a study of non-small cell lung cancer (NSCLC) cell lines, revealed that treatment with methylnaltrexone inhibited invasion of cancer cells (70). In the clinical setting, *post hoc* analysis of two randomized trials, revealed that patients with end stage cancer treated with methylnaltrexone for opioid induced constipation had improved overall survival in contrast to patients who did not receive methylnaltrexone (74).

As previously mentioned, indirect effects of opioids on cancer cells occur *via* the sympathetic nervous system (SNS) and hypothalamic-pituitary-adrenal (HPA) axis (75). Acute opioid administration enhances activity in the periaqueductal grey matter which activates the SNS. The SNS innervates lymphoid organs, such as the spleen, and this activation causes the release of biological amines which reduce splenic lymphocyte proliferation and NK cell cytotoxicity (76). Additionally, prolonged use of opioids increases HPA axis activity and glucocorticoid production, which decrease NK cell cytotoxicity (76). Animal models have shown that this is not a class effect and that it varies between opioid subgroups. These studies have shown morphine and fentanyl to suppress NK cell cytotoxicity whereas buprenorphine does not affect NK cell cytotoxicity (77) and tramadol increases NK cell cytotoxicity, reducing metastasis (78). Differences between opioid subclasses are also evident in clinical trials. However, we could not find any high quality randomised control clinical trials that support these preclinical findings.

Clinical studies investigating the effects of perioperative opioid administration on cancer recurrence displayed conflicting results. Similar to previous data, a more recent retrospective study (2020) comprising of 2,775 patients undergoing surgery for renal cell carcinoma, revealed that higher intraoperative oral morphine milligram equivalent (MME) administration was associated with worse recurrence free survival (RFS) (79). The authors demonstrated that on multivariable analysis, the hazard ratio (HR) was 1.04 per 10 MME (95% CI: 1.01-1.07; P=0.018). Therefore, the trend over the past few decades in experimental and observational studies, has been that the perioperative use of opioids during cancer surgery is associated with negative oncological outcomes. Consequently, this may encourage anaesthesiologists to change their clinical practice in relation to caring for patients undergoing cancer surgery, i.e. by providing an ‘opioid free’ anaesthesia.

However, recent randomised data published in the past few years have not identified such links in large scale clinical practice. In a recent randomised control trial (2021), 146 patients with prostate cancer scheduled for radical prostatectomy were randomised into opioid-free anaesthesia or opioid-based anaesthesia. The authors concluded that intraoperative opioid use did not alter biochemical recurrence free survival in this cohort of patients (80). Similarly, our RCT cited above, of 2,132 women in 13 countries compared regional, (paravertebral blocks and propofol), with general, (sevoflurane and opioid-based analgesia), anaesthesia on breast cancer recurrence. We concluded that regional anaesthesia and the avoidance of opioids did not reduce cancer recurrence after surgery for primary breast cancer compared with general anaesthesia (58). Furthermore, a meta-analysis of thirteen studies regarding perioperative opioids and colorectal cancer indicated that there is no robust evidence to avoid the use of opioids with the primary goal of reducing risk of cancer recurrence (81).

On the other hand it is interesting to note, a more recent observational study suggests a possible beneficial effect of intraoperative opioids on cancer recurrence. A retrospective database study of 1,143 patients with triple negative breast cancer (TNBC), analysed opioid receptor expression patterns in the tumour microenvironment using publicly available bulk and single-cell RNA-sequence data. The investigators identified opioid receptor expression in the TNBC tumours and analysed it alongside its corresponding clinical anaesthesia management and oncologic outcomes. The use of higher doses of intraoperative opioids correlated with improved recurrence free survival but was not significantly associated with improved overall survival (82).

While evidence from laboratory, healthy volunteer, clinical and surgical studies suggest that different opioids variably influence protective anti-tumour immunity, inconsistencies remain in the results of these studies. These may be explained in part by the different methodologies, species, and opioids used, and the dose and duration of their administration. Timing of opioid administration, along with differences in opioid dose and duration of administration, can influence outcome. Large clinical trials have not revealed consistent links between cancer recurrence and perioperative opioid administration. In this growing era of personalised medicine, efforts to differentiate the effects of opioids across cancer subtypes, (and ultimately individual patients), should continue. Given that current data from patients with cancer are inconclusive, categorical recommendations about how acceptable analgesia is best delivered cannot be made and opioids for cancer-related pain will continue to be recommended.

## Ketamine

Ketamine is a phencyclidine derivative that was first synthesised in 1960’s and this racemic compound has been widely adopted in clinical practice. It is used as an induction agent for general anaesthesia and for procedural sedation. In addition, it has potent analgesia properties and is widely used for both acute and chronic pain management. Its anaesthesia and analgesia effects are achieved by acting as a competitive antagonist to N-Methyl-D-Aspartate (NMDA) receptors located in the dorsal horn of the spinal cord (83).

Subanaesthetic doses of ketamine are used for the management of acute perioperative pain. This low dose ranges between 0.5-1mg/kg for a bolus dose, and less than 1.2mg/kg/hr for continuous intravenous administration (84). A Cochrane analysis of the use of intravenous ketamine in the perioperative setting, highlighted that when used as an adjuvant analgesic agent, it reduces postoperative pain scores and opioid consumption (85).

The theoretical concept of ketamine modulating immune function and therefore tumorigenesis dates back to experimental data in the early twenty-first century. These pre-clinical trials demonstrated that ketamine significantly suppressed important pro-inflammatory cytokines that promote tumour production and metastasis; IL-6, IL-8 and TNF-Alpha production (86, 87). In addition, it has been demonstrated that CD4^+^ T-Helper Lymphocyte (Th) cells play a key role in immune protection, these cells are crucial for effective anti-tumour immunity (88). There are two subsets of T-Helper Lymphocytes, Th1 and Th2. In a recent experimental study, Hou et al. (89) highlighted that patients diagnosed with colorectal cancer (CRC) exhibit decreased ratio of Th1/Th2. This imbalance inhibits the hosts immunological response and in turn hastens tumour metastasis. The authors also concluded that morphine further decreases this ratio but the use of ketamine shifted this balance towards Th1, suggesting that ketamine may have a protective immunoregulatory mechanism in patients with CRC (89). Nevertheless, it is worthwhile to note that early experimental data suggests that ketamine significantly suppressed natural killer cell activity and therefore promoted tumour metastasis (90).

A recent randomised control trial (91) disputes this data. The authors randomly assigned 100 patients undergoing colorectal surgery to a control or ketamine group. This clinical trial did not convey any favourable effect on postoperative NK cell activity or diminish pro-inflammatory cytokine levels. The incidence of cancer recurrence or metastasis within two years after surgery were the same between the experimental (Ketamine) and control groups. However, this study was not statistically powered to examine cancer prognosis after surgery as a primary outcome (91). Two recent large retrospective studies in patients with early-stage lung adenocarcinoma (2021) (92) and renal cell carcinoma (2020) (79), found an association between the use of ketamine as an analgesic agent, and reduced perioperative opioid consumption. Furthermore, on multivariable analysis of these retrospective studies, using ketamine as an analgesic adjuvant versus no adjuvant improved the RFS in both renal cell carcinoma (HR = 0.4, 95% CI 0.16-1.00; P=0.050) (79) and in lung adenocarcinoma (HR = 0.44, 95% CI: 0.24-0.80; P=0.007) (92).

The immunomodulatory effects of ketamine may depend on the tumour type, stage and grade. Administration of ketamine as an adjuvant in combination with other opioid sparring analgesia techniques, such as regional anaesthesia and intravenous lidocaine, may also have an influence on immunomodulation. Whether the analgesic effects of ketamine on the observed improved RFS in renal and lung carcinoma, are due to its direct effect on tumour biology or indirect effect (i.e. opioid sparring) remains debatable. This is novel and merits further high quality clinical trials to guide perioperative physicians.

## Dexmedetomidine

Dexmedetomidine is an alpha-2-adrenoceptor agonist drug, and it was first introduced into clinical practice in 1999 as a sedative for mechanically ventilated patients in ICU (93). Pharmacologically, it is D-isomer of medetomidine, a full agonist to alpha-2-adrenergic receptors and in comparison to clonidine, another alpha-2-adrenoreceptor, dexmedetomidine is more selective towards these receptors. Dexmedetomidine has a specificity of 1620:1 (alpha-2: alpha-1), whereas clonidine affinity is 220:1 (alpha-2: alpha-1) (93). It can be administered *via* various routes; intravenous, intranasal, intrathecal and as an adjuvant in peripheral nerve blocks. At present, its clinical application extends beyond the critical care environment. It is now used during the perioperative period to reduce anaesthesia requirements, as a sedative agent, to attenuate the surgical stress response and as an acute analgesic agent. The analgesic mechanism of action of dexmedetomidine is not fully understood but it is thought to produce analgesia by the following pathways (94): 1. Dose-dependent inhibition of C pain fibres, 2. Inhibition of neurotransmission through the dorsal horn of the spinal cord *via* activation of alpha-2-adrergic receptors in the locus coeruleus area of the rostral pons and 3. Promotion of the release of acetylcholine from spinal interneurons. The blunting of systemic sympathetic activation and opioid sparing effects of alpha-2-adrenoceptor agonists (95) are of particular interest in cancer surgery. It is hypothesised that these effects may influence cancer prognosis.

A recent meta-analysis highlighted that intraoperative use of dexmedetomidine may be a favourable analgesic adjuvant in breast cancer surgery, which in turn could reduce both postoperative pain and incidence of postoperative nausea and vomiting (96). Despite this, there is growing concern that its use may negatively impact cancer prognosis. Experimental data have demonstrated that expression of alpha-1 and alpha-2 adrenergic receptors on basal-like breast cancer cells were associated with a poor prognosis (97), and subsequent adrenergic receptor activation by dexmedetomidine may promote proliferation, migration and invasion of breast (98–100), lung (100, 101) and colon (100) cancer cells. However, one study found that dexmedetomidine alone or in combination with propofol had minimal effect on the migration of colorectal cancer cells (102). In addition, recent retrospective data did not demonstrate that intraoperative use of dexmedetomidine was associated with a reduction in recurrence free survival after lung cancer surgery (103) or affect biochemical recurrence and radiological progression following prostate cancer surgery (104). Therefore, the use of adrenergic receptor agonists, notably dexmedetomidine, in cancer surgery could do more harm than good and remains debatable. High quality randomised control trials are warranted before a change of practice is recommended. At present, there are two ongoing randomised control trials (NCT03109990 & NCT03012971: clinicaltrials.gov) which aim to examine overall cancer survival and recurrence in patients receiving an intravenous dexmedetomidine infusion as an analgesic adjuvant versus placebo during cancer surgery.

## Non-Steroidal Inflammatory Drugs (NSAIDs)

NSAIDs are commonly used as analgesics in the perioperative setting and may also provide supplementary anticancer benefits. NSAIDs can be either non-selective, (aspirin, diclofenac, naproxen, ibuprofen, ketorolac), or selective for either the cyclooxygenase 1 (COX1) isoform (ketoprofen) or the COX2 isoform (celecoxib, parecoxib, etodolac, rofecoxib) and have been demonstrated to play an important role in multimodal analgesia for oncological surgery. NSAIDs may prolong the recurrence-free survival of patients after cancer surgery by three distinct mechanisms; first, NSAIDs can reduce the postoperative tumour burden by having a direct effect on cancer cells. For example, celecoxib has been shown to inhibit the formation of surgery-induced metastasis in animal models of colorectal cancer by inhibiting the prostaglandin E2 (PGE2)-glycogen synthase kinase-B catenin pathway (105). Secondly, as inflammation influences the metastatic process, methods of regulating systemic and local inflammatory responses to surgery, may prevent the escape of cancer cells from immunosurveillance in the tumour microenvironment. Lastly, NSAIDs have significant opioid-sparing effects. Opioids have been implicated in postoperative cancer recurrence as discussed previously in this article. In animal models, the use of NSAIDS during surgery has been shown to reduce NK cell numbers and prevent the growth of metastases in murine models (106). By reducing tumour associated inflammation, NSAIDs have also been shown to reduce the extent of angiogenesis and lymphangiogenesis in animal models (107, 108).

Clinical trials indicate that NSAIDs have both local and systemic anti-inflammatory effects. Preoperative use of NSAIDs has been shown to reduce intra-tumoral levels of VEGF expression, lymphangiogenesis, and Treg cell infiltration (109, 110). A study of perioperatively delivered COX2 inhibitors revealed a reduction in prostaglandin levels at the surgical site and in the systemic circulation. Similarly COX2 inhibitors have been shown to suppress increases in systemic catecholamine, cytokine and T-cell levels, and to also buffer the reduction in NK cell counts in the postoperative period (111–115). Data from these prospective clinical studies (2014–2017) suggest an indirect anticancer effect.

There has been considerable effort spent in investigating oncological outcomes related to long term NSAID use prior to, or after diagnosis in cancer patients. Observational studies have shown that regular NSAID use has been associated with improved cancer recurrence rates in colorectal cancer (116) and breast cancer (117). However, perioperative administration of NSAIDs during cancer surgery at analgesic doses have demonstrated variable results in terms of any association with cancer recurrence and overall survival outcomes (118–122). **Table 3** summaries these retrospective studies.

**Table 3:** Selected retrospective studies examining the association between perioperative administration of NSAID and cancer recurrence and overall survival rates.

Author	Year	NSAID	Number of patients	Cancer type	Findings
Forget et al. (118)	2011	Ketorolac	1,111	Prostate cancer	Intraoperative use of Ketorolac did not significantly improve the incidence of biochemical recurrence-free survival rates
Forget et al. (119)	2014	Ketorolac and Diclofenac	720	Breast cancer	Intraoperative use of ketorolac or diclofenac was associated with improved outcomes in cancer recurrence and overall survival rates.
Yeh et al. (120)	2015	Non-specific	15,574	Hepatocellular carcinoma	The use of NSAIDS was associated with a reduced risk of early HCC recurrence within 2 years after liver surgery.
Lee et al. (121)	2016	Ketorolac, ibuprofen, rofecoxib or celecoxib.	1,637	Non-small-cell lung cancer	Perioperative use of NSAID did not significantly improve cancer recurrence and overall survival rates.
Huang et al. (122)	2018	Flurbiprofen and dexamethasone combination	588	Non-small-cell lung cancer	Perioperative combined administration of dexamethasone and flubiprofen was associated with longer survival rates.

Finally, a systematic review on NSAIDs in the oncological surgical population, included studies up to 2017 and concluded that the evidence is equivocal regarding the short-term effects of these analgesic/inflammatory agents on cancer recurrence after cancer surgery (123). Furthermore, two recent prospective RCTs examining these effects have not provided definitive conclusions. A 2019 study comprising of 203 patients scheduled to undergo curative surgery for breast cancer, revealed that a single administration of 30mg of ketorolac preoperatively does not increase disease-free survival in high-risk breast cancer patients. The authors conceded however, that this study was hugely underpowered due to lower recurrence rates than initially anticipated (124). In addition, a 2021 multicentre study of 2639 patients conducted in 160 centres in Germany and the UK, revealed no evidence of a disease-free benefit for 2 years’ treatment with celecoxib compared with placebo, as adjuvant treatment of ERBB2-negative breast cancer. The authors concluded that longer-term treatment or use of a higher dose of celecoxib may lead to a disease-free benefit. Further high-powered clinical trials would be required to further investigate this (125).

## Summary

The hypothesis that anaesthetic and analgesic technique during cancer surgery could influence risk of subsequent recurrence or metastasis has been topical for more than 15 years. Although there is some supportive *in vitro* and *in vivo* experimental data, and also observational clinical data suggesting such an association, only prospective randomised clinical trials can prove a causal link between perioperative analgesia and long-term oncologic outcomes. The first and only large trial available to date has shown robust equivalent findings with regional or volatile general anaesthesia with opioid analgesia. A number of other prospective RCTs evaluating the effect of various analgesic drugs during surgery for cancer resection on disease free survival are ongoing, especially the VAPOR-C trial. These will provide crucial evidence over the coming 5 years which will definitively answer this urgent research question of our time: whether this hypothesis has any meaningful clinical implications for the perioperative care of our cancer resection patients?

## Author Contributions

Conceptualization of this article was performed by DB. Literature search, selection of relevant original investigations for inclusion, and initial writing synthesis: AM and AE. Writing- original draft preparation: AM, AE, and DB. Writing-review and editing: AM, AE, and DB. All named authors contributed to the intellectual content and approved the final submitted version.

## Funding

This research was funded by the division of Anaesthesiology & Perioperative Medicine, Mater University Hospital, Dublin, University College Dublin, Ireland.

## Conflict of Interest

The authors declare that the research was conducted in the absence of any commercial or financial relationships that could be construed as a potential conflict of interest.

## Publisher’s Note

All claims expressed in this article are solely those of the authors and do not necessarily represent those of their affiliated organizations, or those of the publisher, the editors and the reviewers. Any product that may be evaluated in this article, or claim that may be made by its manufacturer, is not guaranteed or endorsed by the publisher.
